# Supplementary material for: Heparin-based hydrogel scaffolding alters the transcriptomic profile and increases the chemoresistance of MDA-MB-231 triple-negative breast cancer cells
Source: Biomater Sci. 2020 Feb 13;8(10):2786–96. doi: 10.1039/c9bm01481k (PMC7497406; doi:10.1039/c9bm01481k)
Supplement: Supplementary file 2 [file BM-008-C9BM01481K-s002.zip › Supplementary File 4/HP-B hydrogel + EGF (s)vControl/Pathways/my_analysis.Gsea.1545201170987/HALLMARK_TGF_BETA_SIGNALING.html]

Details for gene set HALLMARK\_TGF\_BETA\_SIGNALING[GSEA]

|  || Dataset | expr.class.cls#HYDEGF\_versus\_CONTROL.class.cls#HYDEGF\_versus\_CONTROL\_repos |
| Phenotype | class.cls#HYDEGF\_versus\_CONTROL\_repos |
| Upregulated in class | HYDEGF |
| GeneSet | HALLMARK\_TGF\_BETA\_SIGNALING |
| Enrichment Score (ES) | 0.31131324 |
| Normalized Enrichment Score (NES) | 1.1429682 |
| Nominal p-value | 0.25649914 |
| FDR q-value | 0.36607775 |
| FWER p-Value | 0.999 |
Table: GSEA Results Summary

  

Fig 1: Enrichment plot: HALLMARK\_TGF\_BETA\_SIGNALING      
 Profile of the Running ES Score & Positions of GeneSet Members on the Rank Ordered List

  

| PROBE | DESCRIPTION (from dataset) | GENE SYMBOL | GENE\_TITLE | RANK IN GENE LIST | RANK METRIC SCORE | RUNNING ES | CORE ENRICHMENT || 1 | NOG | na |  |  | 314 | 2.357 | 0.0322 | Yes |
| 2 | ENG | na |  |  | 401 | 2.231 | 0.0737 | Yes |
| 3 | SMAD6 | na |  |  | 491 | 2.136 | 0.1130 | Yes |
| 4 | SMAD7 | na |  |  | 606 | 2.006 | 0.1484 | Yes |
| 5 | PPP1R15A | na |  |  | 1051 | 1.653 | 0.1593 | Yes |
| 6 | ID3 | na |  |  | 1178 | 1.582 | 0.1853 | Yes |
| 7 | ID2 | na |  |  | 1377 | 1.479 | 0.2054 | Yes |
| 8 | SKI | na |  |  | 1501 | 1.433 | 0.2285 | Yes |
| 9 | SMURF1 | na |  |  | 1525 | 1.420 | 0.2566 | Yes |
| 10 | SMURF2 | na |  |  | 1621 | 1.385 | 0.2801 | Yes |
| 11 | PMEPA1 | na |  |  | 1964 | 1.277 | 0.2886 | Yes |
| 12 | HIPK2 | na |  |  | 2027 | 1.260 | 0.3113 | Yes |
| 13 | BCAR3 | na |  |  | 3587 | 0.886 | 0.2482 | No |
| 14 | NCOR2 | na |  |  | 3891 | 0.831 | 0.2495 | No |
| 15 | SMAD3 | na |  |  | 6056 | 0.496 | 0.1467 | No |
| 16 | SLC20A1 | na |  |  | 6250 | 0.469 | 0.1463 | No |
| 17 | CDK9 | na |  |  | 6356 | 0.457 | 0.1502 | No |
| 18 | APC | na |  |  | 6402 | 0.450 | 0.1571 | No |
| 19 | ID1 | na |  |  | 6833 | 0.393 | 0.1427 | No |
| 20 | ARID4B | na |  |  | 7169 | 0.349 | 0.1324 | No |
| 21 | FURIN | na |  |  | 7555 | 0.298 | 0.1185 | No |
| 22 | TJP1 | na |  |  | 8311 | 0.208 | 0.0833 | No |
| 23 | LTBP2 | na |  |  | 9080 | 0.117 | 0.0457 | No |
| 24 | ACVR1 | na |  |  | 9142 | 0.110 | 0.0447 | No |
| 25 | TRIM33 | na |  |  | 9224 | 0.100 | 0.0426 | No |
| 26 | SKIL | na |  |  | 9738 | 0.045 | 0.0167 | No |
| 27 | BMPR1A | na |  |  | 10633 | -0.057 | -0.0288 | No |
| 28 | FNTA | na |  |  | 11046 | -0.113 | -0.0480 | No |
| 29 | SMAD1 | na |  |  | 11595 | -0.174 | -0.0730 | No |
| 30 | PPP1CA | na |  |  | 12980 | -0.337 | -0.1384 | No |
| 31 | TGIF1 | na |  |  | 13124 | -0.357 | -0.1385 | No |
| 32 | PPM1A | na |  |  | 13547 | -0.420 | -0.1519 | No |
| 33 | CDKN1C | na |  |  | 13565 | -0.423 | -0.1440 | No |
| 34 | CDH1 | na |  |  | 13575 | -0.423 | -0.1358 | No |
| 35 | TGFB1 | na |  |  | 14260 | -0.515 | -0.1609 | No |
| 36 | JUNB | na |  |  | 15969 | -0.814 | -0.2333 | No |
| 37 | MAP3K7 | na |  |  | 16187 | -0.867 | -0.2268 | No |
| 38 | BMPR2 | na |  |  | 16383 | -0.919 | -0.2180 | No |
| 39 | UBE2D3 | na |  |  | 16494 | -0.946 | -0.2043 | No |
| 40 | TGFBR1 | na |  |  | 16855 | -1.043 | -0.2016 | No |
| 41 | KLF10 | na |  |  | 16932 | -1.064 | -0.1836 | No |
| 42 | HDAC1 | na |  |  | 17151 | -1.128 | -0.1718 | No |
| 43 | WWTR1 | na |  |  | 17497 | -1.258 | -0.1639 | No |
| 44 | FKBP1A | na |  |  | 17786 | -1.373 | -0.1506 | No |
| 45 | CTNNB1 | na |  |  | 17952 | -1.465 | -0.1291 | No |
| 46 | RHOA | na |  |  | 17958 | -1.466 | -0.0991 | No |
| 47 | XIAP | na |  |  | 18281 | -1.662 | -0.0817 | No |
| 48 | IFNGR2 | na |  |  | 18361 | -1.722 | -0.0503 | No |
| 49 | BMP2 | na |  |  | 18629 | -1.962 | -0.0238 | No |
| 50 | RAB31 | na |  |  | 19005 | -2.597 | 0.0101 | No |
Table: GSEA details [plain text format]

  

Fig 2: HALLMARK\_TGF\_BETA\_SIGNALING      
 Blue-Pink O' Gram in the Space of the Analyzed GeneSet

  

Fig 3: HALLMARK\_TGF\_BETA\_SIGNALING: Random ES distribution      
 Gene set null distribution of ES for **HALLMARK\_TGF\_BETA\_SIGNALING**
